# Impact of Juvenile Idiopathic Arthritis Associated Uveitis in Early Adulthood

**DOI:** 10.1371/journal.pone.0164312

**Published:** 2016-10-10

**Authors:** Anne-Mieke J. W. Haasnoot, Lenneke A. Vernie, Aniki Rothova, Patricia v. d. Doe, Leonoor I. Los, Nicoline E. Schalij-Delfos, Joke H. de Boer

**Affiliations:** 1 Department of Ophthalmology, Utrecht University Medical Center, Utrecht, The Netherlands; 2 Department of Ophthalmology, Erasmus Medical Center, Rotterdam, The Netherlands; 3 Department of Ophthalmology, Groningen University Medical Center, Groningen, the Netherlands; 4 W.J. Kolff Institute, Graduate School of Medical Sciences, University of Groningen, Groningen, the Netherlands; 5 Department of Ophthalmology, Leiden University Medical Center, Leiden, The Netherlands; Oregon Health and Science University, UNITED STATES

## Abstract

**Background:**

Typically juvenile idiopathic arthritis (JIA)-associated uveitis (further referred as ‘JIA-uveitis’) has its onset in childhood, but some patients suffer its, sometimes visual threatening, complications or ongoing disease activity in adulthood. The objective of this study was to analyze uveitis activity, complications and visual prognosis in adulthood.

**Methods:**

In this multicenter study, 67 adult patients (129 affected eyes) with JIA-uveitis were retrospectively studied for best corrected visual acuity, visual fields, uveitis activity, topical/systemic treatments, ocular complications, and ocular surgeries during their 18^th^, 22^nd^ and 30^th^ year of life. Because treatment strategies changed after the year 1990, outcomes were stratified for onset of uveitis before and after 1990.

**Results:**

Sixty-two of all 67 included patients (93%) had bilateral uveitis. During their 18^th^ life year, 4/52 patients (8%) had complete remission, 28/52 (54%) had uveitis activity and 37/51 patients (73%) were on systemic immunomodulatory treatment. Bilateral visual impairment or legal blindness occurred in 2/51 patients (4%); unilateral visual impairment or legal blindness occurred in 17/51 patients (33%) aged 18 years. The visual prognosis appeared to be slightly better for patients with uveitis onset after the year 1990 (for uveitis onset before 1990 (n = 7) four patients (58%) and for uveitis onset after 1990 (n = 44) 13 patients (30%) were either visual impaired or blind). At least one ocular surgery was performed in 10/24 patients (42%) between their 18^th^ and 22^nd^ year of life.

**Conclusions:**

Bilateral visual outcome in early adulthood in patients with JIA-uveitis appears to be fairly good, although one third of the patients developed one visually impaired or blind eye. However, a fair amount of the patients suffered from ongoing uveitis activity and needed ongoing treatment as well as surgical interventions. Awareness of these findings is important for ophthalmologists and rheumatologists treating patients with JIA-uveitis, as well as for the patients themselves.

## Introduction

Chronic anterior uveitis is the most common extra-articular manifestation of juvenile idiopathic arthritis (JIA), a serious disease starting prior to the age of 16 years.[1] During the course of, or preceding JIA, uveitis occurs in 10% up to 45% of the patients, depending on the specific subtype of JIA.[1–3] Typically JIA-associated uveitis (further referred as ‘JIA-uveitis’) has its onset in childhood, but some patients suffer its, sometimes visual threatening, complications or ongoing disease activity in adulthood. Common complications include band keratopathy, posterior synechiae, cataract, secondary glaucoma and cystoid macular edema (CME).[4–6] However, previous clinical studies seldom investigated a follow-up of more than seven years.[7–11] Previously, we reported on uveitis activity in childhood and puberty and this study revealed a flare up of uveitis during puberty.[12] Little is known about JIA-uveitis activity in adulthood, but there are suggestions that uveitis activity may persist in adulthood in 30–63% of the patients. Reported outcomes differ between various studies due to broad follow-up ranges.[12–18] Therefore, the course of uveitis activity and outcome in adulthood remain unclear. Furthermore, treatment strategies changed drastically with the upcoming of methotrexate (MTX) around 1990 and later anti-TNFα therapy around the year 2000.[19–22] Also, ophthalmologic screening protocols for patients with JIA were introduced around 1990.[23–25] The objective of this study was to analyze uveitis activity and its complications and visual prognosis in adults with JIA-uveitis and to compare patients with an onset of uveitis before and after the year 1990.

## Methods

A retrospective chart review of 67 patients with JIA-associated chronic anterior uveitis who had reached the age of 18 years, examined at the tertiary centers University Medical Center of Utrecht, Rotterdam, Leiden and Groningen during the period of 1974 to 2015, was performed. The majority of the included patients were of European descent. In 12 patients, follow-up data at the tertiary centers were not complete, therefore these patients signed an informed consent form in order to make a copy of the ophthalmologist’s patient’s medical chart from general hospitals, where these patients had also been treated. These charts were additionally reviewed. This study was approved by the Institutional Review Board of the Utrecht University Medical Center and is in compliance with Helsinki principles. The Review Board agreed that patients’ informed consent was not necessary, since the data were analyzed anonymously.

JIA was diagnosed and classified by a pediatric rheumatologist according to the criteria of the International League of Associations for Rheumatology (ILAR) or by former criteria, such as the European League Against Rheumatism (EULAR).[26,27] The uveitis diagnosis was made by an ophthalmologist according to the recommendations of the International Uveitis study group.[28]

The following demographic and disease characteristics were documented for every patient: date of birth, gender, JIA subtype, laterality of the uveitis, age at onset of arthritis, age at onset of uveitis, uveitis onset before 1990 or after 1990, the presence of anti-nuclear antibodies (ANA) and the presence of Human Leukocyte Antigen B-27 (HLA-B27). Furthermore, the occurrence of synechiae and band keratopathy and patients’ age at onset of these complications were noted, as well as the cataract and glaucoma surgeries being performed, and the age at which these were done. Finally, the subsequent data were collected at fixed time-points, namely during the year in which the patients became 18, 22 and 30 years old: activity of uveitis, use of topical corticosteroids, use of intraocular pressure (IOP) lowering medication, use of systemic immunomodulatory treatment (IMT), occurrence of complications like CME, papillitis, and hypotony (defined as an IOP <6mmHg in at least two consecutive visits), best corrected visual acuity (BCVA) measured with Snellen charts and visual field defects based on examination with the Rodenstock Peritest or the Humphrey Field Analyzer. When BCVA was measured more times during a year, the best BCVA of the age year was used for analyses.

Patients diagnosed with uveitis after the age of 16 years and patients with a JIA subtype other than juvenile oligoarthritis, extended oligoarthritis or polyarthritis (rheumatoid factor negative) were excluded. Uveitis was considered active when there were at least 1+ cells in the anterior chamber, as determined by the grading system of the Standardization of Uveitis Nomenclature (SUN) working group, at least at one visit during the year of the fixed time-point.[29] Remission was defined as inactive disease for ≥ three months after discontinuing all treatments for eye disease (SUN). Systemic IMT was noted when used for more than six consecutive months and included MTX, corticosteroids, adalimumab, infliximab, other disease modifying anti-rheumatic drugs (mycophenolate mofetil, azathioprine or cyclosporine), other biologicals (tocilizumab or etanercept) or other anti-rheumatic drugs (hydroxychloroquine or sulfasalazine). Starting dose of MTX was 10–15 mg/m^2^ body surface once a week, with a maximum of 20 mg/m^2^, and starting dose of oral prednisone was generally 1mg/kg body weight. If compliance with the usage of medication was reported in the patient’s medical chart, these data were used. Changes in medication, uveitis activity and complications arising within a period of two months after surgery were not included. Measurements of visual acuity were only included for analyses if uninfluenced by or corrected for refractive errors (BCVA).

The database was built per patient, with additionally included information per eye for BCVA, visual field and ocular surgeries. Visual outcome was classified into three groups, based on the criteria of the SUN working group: visual acuity better than 20/50 was defined as no visual impairment, visual acuity equal to or less than 20/50 was defined as visual impairment and visual acuity equal to or less than 20/200 was defined as legal blindness.[29] If patients had a visual field of 10° or less they were also classified as ‘legal blindness’, according to the criteria for visual impairment of the World Health Organization.[30] Data on visual ability (visual acuity in combination with visual field outcome) per eye were combined to analyze the visual impairment per patient, primarily using data of the best eye to determine the visual outcome. If data of the worst eye was used, this is explicitly mentioned.

To study the course of the BCVA, Snellen visual acuity values were transferred to LogMar. When patients had a visual field of ≤10°, the same LogMar visual acuity as for light perception was used (LogMar = 2.90). For patients who had no light perception we used a LogMar visual acuity of 3.20. The reported BCVA’s in this manuscript are reported on a Snellen scale and are based on per eye analyses.

Statistical analysis was performed using SPSS 21.0 for Windows (IBM Corporation, Armonk, New York, USA). Normality was tested using the Shapiro-Wilk test. Medians combined with a range were given for not normally distributed variables. A Chi-squared test or Fisher’s exact test was performed to compare categorical data and a Mann-Whitney U-test was done to compare medians across groups. To analyze the course of the disease in patients of whom data was available at 18 and 22 years of age and at 18, 22 and 30 years, the McNemar test was used to compare dichotomous variables. To compare the visual acuity in these patients, Snellen BCVA was log transformed and median BCVA’s (with interquartile range (IQR)) were compared by the Wilcoxon signed rank test.

## Results

### General characteristics of study population

Sixty-seven patients in total were included (129 affected eyes) and 62 (93%) had bilateral uveitis. Characteristics of the study population at the age of 18, 22 and 30 years (n = 52, 26 and 13 respectively) with uveitis onset before 1990 and after 1990 are shown in Table 1. All data at 30 years were from patients with uveitis onset before 1990. There were no statistically significant differences for baseline characteristics after stratifying for uveitis onset before and after the year 1990.

**Table 1:** Characteristics of patients with juvenile idiopathic arthritis associated uveitis at the age of 18, 22 and 30 years.

			< 1990			≥ 1990		
Characteristics		Total	18 years	22 years	30 years	18 years	22 years	30 years
Total no. patients		67	7	8	13	45	18	0
Total no. eyes		129	14	16	26	86	36	NA
Bilateral uveitis no. patients (%)		62 (93)	7 (100)	8 (100)	13 (100)	41 (91)	18 (100)	NA
Female, no. patients (%)		50 (75)	6 (86)	7 (88)	12 (92)	30 (67)	13 (72)	NA
Median age (y) at onset uveitis (range)		5.2 (1.2–14.6)	4.1 (3.0–9.3)	4.5 (3.0–9.3)	4.9 (3.0–9.3)	5.3 (2.6–14.6)	5.2 (2.6–12.7)	NA
Median age (y) at onset arthritis (range)		3.5 (0.8–16.1)	3.0 (0.8–7.4)	2.9 (0.8–7.4)	3.0 (0.8–7.4)	3.8 (0.9–16.1)	4.7 (1.0–12.7)	NA
Interval (y) arthritis-uveitis, median (range)		0.5 (-9.8–13.3)	0.7 (-3.4–6.5)	1.6 (-3.4–6.5)	0.7 (-3.4–6.5)	0.4 (-9.8–13.3)	0.5 (-6.7–6.0)	NA
Median duration (y) of uveitis (range)		NA	13.9 (8.7–15.0)	17.5 (12.7–19.0)	25.1 (20.7–27.0)	12.7 (3.4–15.4)	16.9 (9.3–19.4)	NA
Arthritis onset before uveitis, no. patients (%)		48 (74)	5 (83)	6 (86)	9 (82)	30 (67)	13 (72)	NA
JIA subtype, no. patients (%)	Oligoarthritis	35 (52)	4 (57)	4 (50)	5 (38)	28 (62)	9 (50)	NA
	Extended oligoarthritis	9 (13)	0 (0)	0 (0)	1 (8)	7 (16)	5 (28)	NA
	Polyarthritis	15 (22)	1 (14)	1 (13)	2 (16)	8 (18)	4 (22)	NA
	Unknown	8 (12)	2 (29)	3 (37)	5 (38)	2 (4)	0 (0)	NA
ANA, no. patients	Total	64	7	8	12	44	18	NA
	Positive (%)	52 (81)	6 (86)	7 (88)	11 (92)	37 (84)	15 (83)	NA
HLA-B27, no. Patients	Total	28	1	2	5	18	8	NA
	Positive (%)	5 (18)	0 (0)	0 (0)	1 (20)	3 (17)	0 (0)	NA

JIA = juvenile idiopathic arthritis; ANA = antinuclear antibodies; HLA-B27 = human leukocyte antigen B27; NA = not applicable. Per age-group statistical analyses were performed comparing patients with an uveitis diagnosis before (<) or after (≥) 1990 by the Fisher exact test and Mann-Whitney U test. No statistically significant differences were found. In the group with an uveitis diagnosis <1990, more data were available at age 30 years than at age 18 or 22 years. Because of the retrospective character of the study, more data were missing at the latter two time-points.

### Visual impairment and visual acuity

**Fig 1:**
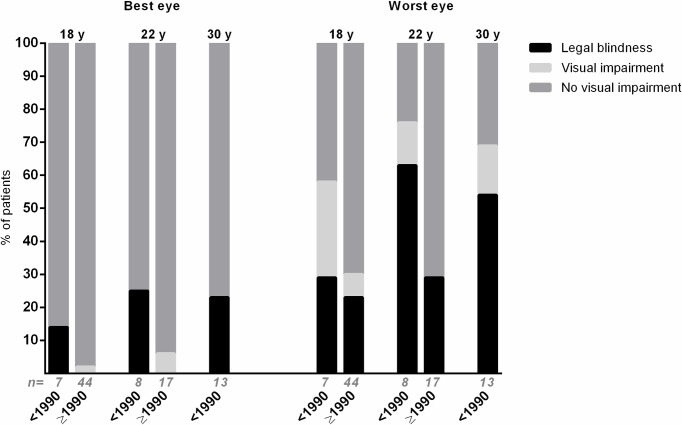
Visual impairment of patients with juvenile idiopathic arthritis associated uveitis. Proportion of patients with juvenile idiopathic arthritis associated uveitis with no visual impairment, visual impairment or legal blindness of the best (left) and worst (right) eye at the age of 18, 22 and 30 years, with uveitis onset before (<) 1990/after (≥) 1990. Visual acuity better than 20/50 was defined as no visual impairment, visual acuity equal to or less than 20/50 was defined as visual impairment and visual acuity equal to or less than 20/200 was defined as legal blindness.[29] If patients had a visual field of 10° or less they were also classified as ‘legal blindness’, according to the criteria for visual impairment of the World Health Organization.[30]. Statistical analyses comparing the situation <1990 and ≥1990 were derived from the Fisher exact test, there were no statistically significant differences. ‘n = ‘ = total number of patients included in the bar.

Data of the visual ability at the different ages are shown in Fig 1. At the age of 18 years, 2/51 patients (4%) were visually impaired or legally blind. One was diagnosed before 1990 and one after 1990. Seventeen out of 51 patients (33%) had at least one eye which had a visual acuity of 20/50 or worse and/or a visual field of ≤10° of which 13/17 patients were diagnosed after 1990 (which represents 30% of all 44 patients diagnosed after 1990) and 4/17 patients were diagnosed before 1990 (which represents 58% of all seven patients diagnosed before 1990).

Of all patients with an impaired visual ability of at least one eye, 10 out of 17 (59%) were diagnosed with uveitis before arthritis versus five out of 33 (15%) of the patients with a good visual ability (*P* = .001). Also at the age of 22 years, the two patients (100%) with visual impairment of the best eye were diagnosed with uveitis before arthritis, compared to three out of 22 patients (14%) with normal visual acuity (*P* = .036).

No differences were found for gender, uveitis onset before 1990 or after 1990, or total number of ocular surgeries since uveitis onset.

The course of visual acuity could be measured for 23 patients with follow-up data at both 18 and 22 years. The median BCVA between 18 and 22 years (n = 23 patients) appeared to be stable. Sixteen of these patients (70%) were diagnosed after 1990. Six patients had follow-up data at all age years (18, 22 and 30 years) and had a median BCVA of one eye of 20/63 (IQR 20/20-0.25/200) at the age of 18 years and 2/200 (IQR 20/22-0.25/200) at the age of 30 years (*P* = .465) and for the other eye a median BCVA of 20/25 at 18 years (IQR 20/16-20/100) and 20/40 at 30 years (IQR 20/25-0.02/200; *P* = .116). All these six patients were diagnosed before the year 1990.

### Uveitis activity and treatment

**Fig 2:**
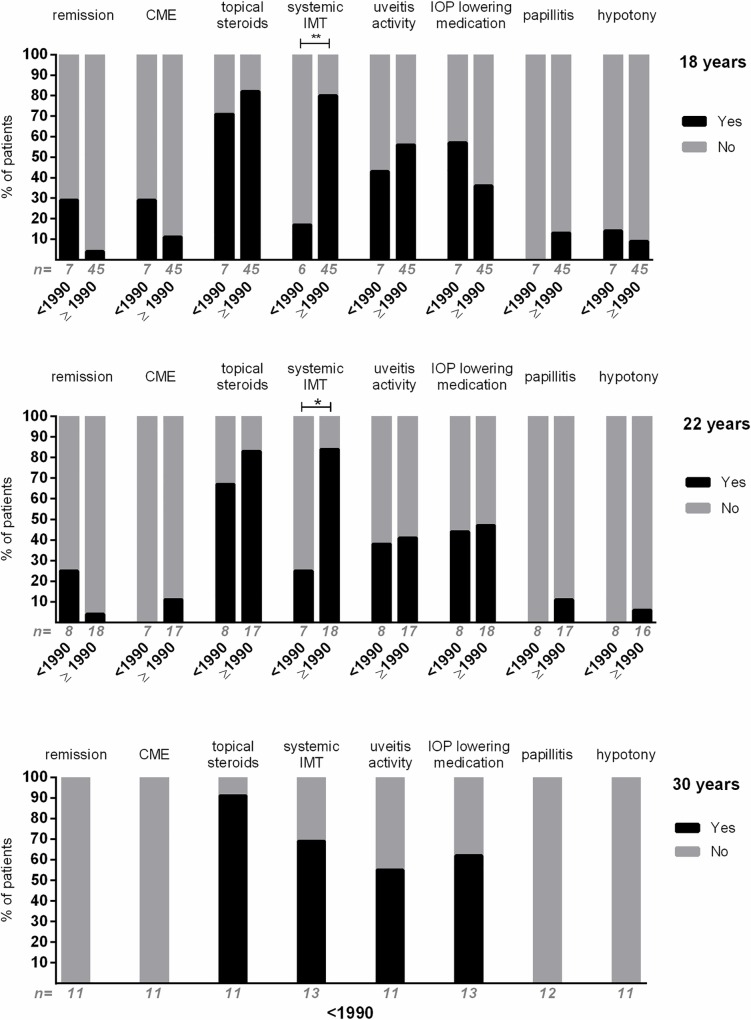
Uveitis activity, treatment and complications of patients with uveitis associated with juvenile idiopathic arthritis in adulthood. Outcomes of patients with uveitis onset before (<) and after (≥) the year 1990. At the top: outcomes at age 18; middle: age 22 years; bottom: age 30 years. Note that all data at age 30 years were from patients with uveitis onset before the year 1990. Remission was defined as inactive disease for ≥ three months after discontinuing all treatments for eye disease.[29] Systemic immunomodulatory treatment (IMT) was defined ‘Yes’ when used for more than six months. Uveitis activity was defined ‘Yes’ when there were at least 1+ cells in the anterior chamber, as determined by the grading system of the Standardization of Uveitis Nomenclature (SUN) working group.[29]. CME = cystoid macular edema; IOP = intraocular pressure. *: P < .05; **: P < .005. ‘n = ‘ = total number of patients included in the bar.

Of the 52 patients with available data at the age of 18 years, four patients (8%) were in remission during the entire year (Fig 2). Almost half of the patients (n = 28/52, 54%) had an episode of active uveitis during their 18^th^ life year, 37/51 patients (73%) were on systemic IMT and 42/52 (81%) used topical steroids. Four of the 37 (11%) 18 year-old patients on systemic IMT had no uveitis activity and did not use topical steroids (all diagnosed ≥1990). All others in the group ‘without remission’ were at least on topical corticosteroids or had uveitis activity.

The majority of 18 year-old patients diagnosed after 1990 were on systemic IMT (n = 36/45, 80%), in contrast to the patients diagnosed before 1990, where only 1/6 patients (17%) was on systemic IMT during the 18^th^ life year (*P* = .004). A comparable result was found at the age of 22 years (*P* = .017, Fig 2). No other statistically significant differences were found for uveitis activity or treatment between the patients diagnosed before and after 1990.

**Fig 3:**
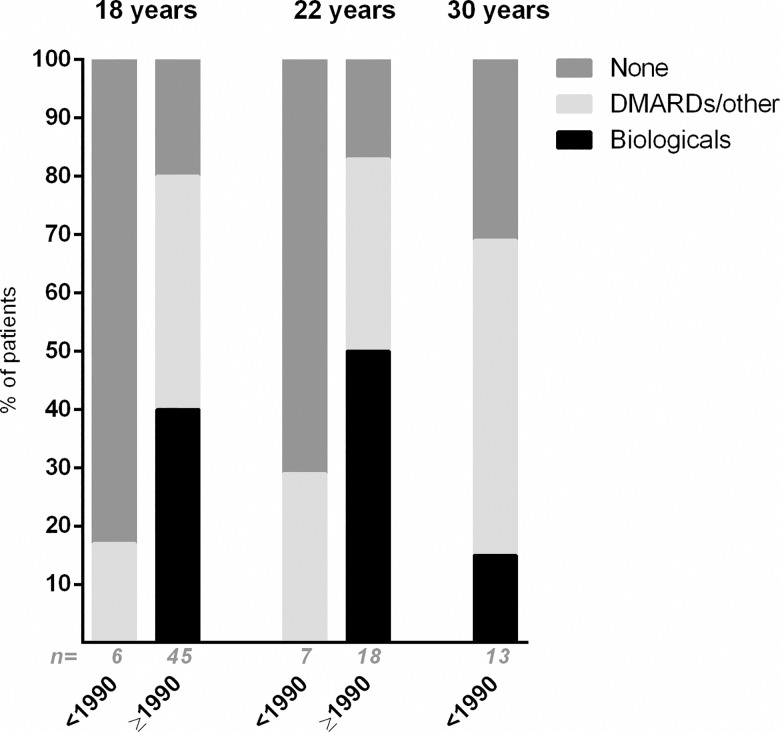
The use of systemic immunomodulatory treatment in patients with juvenile idiopathic arthritis associated uveitis. Treatment at the age of 18, 22 and 30 years with uveitis onset before (<) and after (≥) the year 1990. DMARD = disease modifying anti-rheumatic drugs. A biological was always given combined with a DMARD (in the figure noted as ‘Biologicals’), a DMARD/other drugs were usually given as combination therapy. ‘n = ‘ = total number of patients included in the bar. The exact treatment, with number of patients using this treatment in brackets, was: Treatment 18 years, <1990: Corticosteroids (n = 1). Treatment 18 years, ≥ 1990: Corticosteroids (n = 1), Methotrexate (n = 31), Mycophenolate motefil (n = 2), Azathioprine (n = 2), Cyclosporine (n = 1), Adalimumab (n = 16), Etanercept (n = 1), Infliximab (n = 2). Treatment 22 years, < 1990: Corticosteroids (n = 1), Methotrexate (n = 1).Treatment 22 years, ≥ 1990: Corticosteroids (n = 2), Methotrexate (n = 9), Mycophenolate motefil (n = 3), Azathioprine (n = 1), Hydroxychloroquine (n = 2), Adalimumab (n = 4), Etanercept (n = 2), Tocilizumab (n = 2), Infliximab (n = 1).Treatment 30 years, <1990: Corticosteroids (n = 4), Methotrexate (n = 5), Mycophenolate motefil (n = 1), Hydroxychloroquine (n = 1), Cyclosporine (n = 1), Sulfasalazine (n = 1), Tocilizumab (n = 1), Etanercept (n = 1).

The different treatment strategies are presented in Fig 3. The figure shows that patients diagnosed after 1990 were treated with biologicals more often than patients diagnosed with uveitis before 1990.

### Course of complications and surgery

Ten of the 24 patients (42%) with follow-up data available at the age of 18 as well as at the age of 22 years underwent surgery between their 18^th^ and 22^nd^ year of age. Three/24 patients (14%) underwent cataract surgery and 9/24 (38%) underwent glaucoma surgery between their 18^th^ and 22^nd^ birthday.

Seventeen/24 patients (71%) had already had surgery by the time they became 18 years old (6/24 patients had had surgery for cataract and glaucoma, 10/24 for cataract only, 1/24 for glaucoma only). Four/24 patients (17%) had their first surgery after the age of 18. Three/24 patients (13%) never underwent surgery before their 22^nd^ life year. Note that all of these 24 patients had bilateral uveitis and the majority of patients (n = 17/24, 71%) was diagnosed with uveitis after 1990.

**Fig 4:**
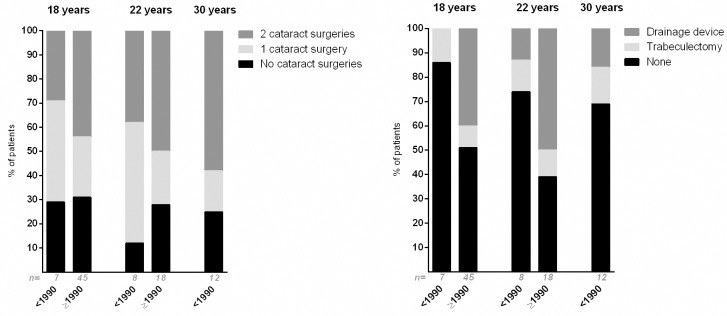
Cataract and glaucoma surgery of patients with uveitis associated with juvenile idiopathic arthritis in adulthood. Proportion of patients who had undergone cataract (left) and glaucoma (right) surgery by the time they were 18, 22 and 30 years old, with uveitis onset before (<) and after (≥) the year 1990. Many patients had multiple glaucoma surgeries; in this figure patients with at least one glaucoma surgery versus no glaucoma surgeries are presented. Some of the patients who received a drainage device, also had a trabeculectomy in their medical history and are here included in the ‘Drainage device’ group. There were no statistically significant differences between the groups with uveitis onset before and after 1990. ‘n = ‘ = total number of patients included in the bar.

Fig 4 presents the number of cataract surgeries and type of glaucoma surgeries performed in the complete study population. Though not statistically significant, these results suggest that patients underwent glaucoma surgery more often when having an uveitis onset after 1990.

None of the patients developed posterior synechiae between the age of 18 and 22 years, though one patient (4%) developed band keratopathy.

Active inflammation in the anterior chamber was less frequent at the 22^nd^ life year (8/23, 35%) compared to the 18^th^ life year (15/23, 65%; *P =* .*039*). No differences were found for complete remission of uveitis, presence of CME, use of topical steroids, use of systemic IMT, use of IOP lowering medication, papillitis or hypotony.

## Discussion

Our results document that JIA is not solely a disease of childhood, but that its activity, accompanied use of systemic medications, complications and additional visual loss continues in early adulthood. Only 4% of the JIA-uveitis patients were in remission at 18 years of age.

Previous literature, as presented in Table 2, described patients with JIA-uveitis in adulthood, but broad ranges of follow-up make it difficult to interpret their results. In our study we studied different outcome measurements at fixed time-points. Further, to get a good impression of the visual ability of the patients, visual acuity cannot be seen apart from visual field outcomes. Only one previous study of 12 adult JIA-uveitis patients also studied visual field outcomes.[14]

**Table 2:** Juvenile idiopathic arthritis associated uveitis in adulthood, a literature overview.

Author	Nr patients	Mean/median age years (range)	Follow-up	Visual acuity	Definition active uveitis	Uveitis activity	Complications, treatment
Packham et al 2002[13]	54	35 (19–78) (= age of total JIA group with 246 patients)	Situation at the end of follow-up.	NA	Not defined	NA	66% of the patients had glaucoma during follow-up, 55% had cataract, 69% had had eye-surgery (not specifically during adulthood).
Zak et al 2003[14]	12	32 (22–49)	Situation at the end of follow-up.	Studied visual field outcomes.^a^ 1 patient with bilateral severe visual field loss. 1 patient with unilateral VA <0.02.	Not defined	NA	Almost all patients had anterior segment findings as sequelae.
Ozdal et al 2005[15]	18 (30 eyes)	30 (18–48)	Situation at the end of follow-up. Minimum follow-up = 2 years.	9 patients (50%) had a BCVA <20/150 of at least one eye.	Presence of cells or keratic precipitates, with or without flare.	19 (63%) of the eyes had active uveitis.	3 eyes with phtisis. 73% (22 eyes) had cataract extraction, 4 eyes (13%) had glaucoma surgery by drainage device or trabeculectomy (not specifically during adulthood). 11 patients (61.1%) required the use of a systemic immunosuppresive agent. 16/18 patients were on topical steroids.
Kotaniemi et al 2005[16]	19	24 (22–26)	One consult for evaluation.	100% binocular normal BCVA, 3 patients had unilateral BCVA <0.1.	Use of topical corticosteroids and/or at least 3 cells in the anterior chamber.	8 patients had active uveitis.	4 patients had glaucoma, 5 had cataract. 53% of the patients were on systemic IMT. 10/19 patients used treatment (systemic and/or topical).
Camuglia et al 2009[17]	17	30 (21–43)	Situation at the end of follow-up.	20% of the eyes had visual loss up to 6/12, 13.3% of the eyes had visual loss up to 6/60.	Not defined	NA	53% of the patients (9 patients, 13 eyes) had new complications of cataract or glaucoma after their 16th birthday. Two eyes had glaucoma surgery and 10 eyes had cataract surgery after the age of 16. 30% of the patients had synechiae during their uveitis course. 10/17 patients used systemic treatment, all patients had topical treatment.
Skarin et al 2009[35]	55	NA	Follow-up at 24 years (range 18–46) after uveitis onset.	NA	Not defined	49% of the 55 patients had signs of active uveitis or were receiving topical corticosteroids.	12 patients (33%) had glaucoma, 28 patients (78%) had cataract.
Oray et al 2016[18]	77 (135 eyes)	29.7 (±11)	Situation at the end of follow-up.	37 eyes (28%) had a visual acuity of ≤20/50. 20 eyes (15%) had a visual acuity of ≤20/200.	≥0.5+ cells in the anterior chamber.	78 eyes (58%).	Ocular surgery in 68 eyes. 13 patients (17%) were treated with conventional IMT (e.g. MTX), 52 patients (68%) were treated with biologicals. At least one complication in 95 eyes (72%).

This table describes all previous literature on the course of juvenile idiopathic arthritis associated uveitis in adulthood.

^a^This was the only study which described visual field outcomes.

NA = not applicable; IMT = immunomodulatory treatment; MTX = Methotrexate.

Our study showed a trend towards a slightly better visual outcome for patients diagnosed after 1990 than for patients diagnosed before 1990. Also, a higher percentage of patients diagnosed after 1990 used systemic IMT, which suggests an improvement of visual outcome due to novel and more intensive immunomodulating treatment strategies since 1990.[19–22] It is likely that the use of ophthalmologic screening guidelines for patients with JIA has also contributed to improved visual outcome.[23–25] Furthermore, the awareness of glaucoma increased during the last decades with earlier and improved interventions and more prevention of severe glaucomatous visual field loss, as also displayed by Fig 4.[31,32] Also, cataract extractions are nowadays performed at an earlier stage, preventing development of amblyopia. Previous literature described a delay of cataract formation in patients treated with MTX, which means that, since the introduction of MTX as a treatment for JIA-uveitis, children have less chance to develop cataract at an age vulnerable for developing amblyopia.[33]

Better visual outcome in relation to systemic treatment was also shown by the study of Gregory et al.[4] Unfortunately, we could not correlate use of systemic IMT to visual outcome, since our study had data at three time-points rather than continuous data over the years. Additionally, the prescription of IMT might have been influenced by uveitis as well as arthritis activity. Anyhow, only four patients were solely on IMT without uveitis activity or topical steroids for their uveitis, suggesting that most patients in this study received systemic IMT at least partially for their uveitis.

Though our results suggest that most patients with JIA-uveitis have a fairly good binocular visual prognosis, with a rather stable visual outcome during the adolescent years, about one third of the patients with uveitis onset after the year 1990 had at least one visually impaired eye (Fig 1). In line with previous literature, risk factors for an impaired visual outcome were onset of uveitis before arthritis and lower age at onset of uveitis.[34] Because of the relatively small sample size in our patient groups, we cannot exclude that other factors which were described previously, such as male gender, are also possible risk factors for visual impairment.[6]

Despite the slight improvement of visual acuity and the increased use of systemic IMT after 1990, uveitis in early adulthood was active in approximately half of the patients. This is an important finding to be aware of, since a previous study described uveitis activity to be associated with an increased risk of visual loss.[4] It is extremely important for ophthalmologists to keep a close eye on these patients and treat them accurately, to prevent them from more bi- or unilateral visual impairment due to uveitis activity in their future lives. A longer follow-up and a larger cohort are necessary to be able to document their visual functioning over the years.

Our study cohort presents a representative JIA-uveitis population, as the male-to-female ratio, median age of arthritis onset and presence of ANA are similar to previous literature.[12,14–17,35,36]

Because of its retrospective character our study has limitations. There might have been a tertiary referral bias, since patients were selected from a cohort of tertiary centers. Though, this was minimized as much as possible by retrieving medical charts from patients being followed also by their own ophthalmologists outside the tertiary centers. Therefore, the percentage of patients with active uveitis and/or complications during adulthood might be lower than presented in this study. The sample size of patients evaluated at the age of 30 years is also limited, and moreover, the data of these patients at their 18^th^ and 22^nd^ year of life were not available, because it was not possible to retrieve their old medical charts.

Overall results on visual outcomes in this study are quite promising, with an improvement in visual acuity, probably also due to systemic IMT. Several studies have shown that that the introduction of MTX and anti-TNFα therapies have been important in the prevention of complications or even the occurrence of uveitis in JIA patients.[4,37] But these treatment strategies might have significant impact on the quality of life of patients. For example, use of systemic IMT will result in more visits to the ophthalmologists or rheumatologists, also, these young adult women might wish to become pregnant which conflicts with the use of most immunosuppressive medications. For future research it would be interesting to investigate the effect of the use of systemic IMT on these aspects, including quality of life of these adult patients.

In conclusion, the results of this study imply that binocular visual outcome in adulthood is fairly good in most patients with JIA-uveitis and that binocular visual ability seems to be stable in the first years after puberty. Despite the apparent improvement in visual prognosis in the last decades, still up to 30% of all adult JIA-uveitis patients have developed severe visual impairment or even blindness of at least one eye. Also, uveitis activity is continuing in early adulthood, and the majority of patients need ongoing treatment. Additionally, uveitis associated complications still arise in early adulthood and a relevant proportion of the patients need cataract or glaucoma surgery during adulthood. Awareness of these findings is important for ophthalmologists and rheumatologists treating patients with JIA-uveitis, as well as for the patients themselves, in order to prevent these patients from becoming visually disabled even many years after their typical childhood onset of this disease.
